# A commentary on ‘Breast-conserving surgery is associated with a lower incidence of suicide among females with breast cancer in the United States: a population-based retrospective cohort study’

**DOI:** 10.1097/JS9.0000000000001484

**Published:** 2024-04-22

**Authors:** Clara Briceño-Morales, Ximena Briceño-Morales

**Affiliations:** a Universidad Tecnológica de Pereira, Pereira, Risaralda; b Universidad Nacional de Colombia, Bogotá, Colombia

*Dear Editor,*

We read with great interest the recent article published by Guo *et al*. titled ‘Breast-conserving surgery is associated with a lower incidence of suicide among females with breast cancer in the United States: a population-based retrospective cohort study’^1^. Using a population-based retrospective cohort, the authors assessed the risk of dying by suicide among breast cancer survivors, comparing breast-conserving surgery (BCS) and mastectomy. This study, which included more than 1 million women, 56.5% with BCS and 36.1% with mastectomy, revealed that patients that had mastectomy showed significantly higher suicide mortality compared to the general population: standardized mortality rate (SMR) of 1.18 (95% CI 1.05–1.33), while there was no significant difference in the suicide rate between patients with BCS and the general population: SMR 0.92 (95% CI 0.83–1.02). Multivariate Cox analysis revealed that BCS, compared with mastectomy, was associated with a significantly lower risk of suicide in women with breast cancer (95% CI 0.68–0.95; *P*=0.009).

We want to thank the authors for providing such valuable evidence on issues related to the mental health of cancer patients, which tend to be underestimated in medicine. Additionally, we would like to make some comments extrapolated to our context. According to the Bulletin published in October 2023 by the Gender Sub-directorate of the Colombian National Planning Department, unlike the global trend but in accordance with the inclination of the continent, the incidence of suicide in Colombia has shown an increase of 22.8% between 2015 and 2022. Although the risk of suicide among cancer patients has been poorly studied in our country, both mental and physical illnesses could be triggers for suicidal situations^2^. Taking into account that breast cancer is the most frequently diagnosed neoplasm in Colombian women, with an annual incidence calculated at 50.7 per 100 000 women^3^ and a standardized prevalence of 329.16 for every 100 000 women^4^, it would be expected that this pathology represents one of the many physical conditions that eventually lead Colombian women to suicide. According to official information from the National Cancer Institute, a Colombian government entity that works in many areas for ‘cancer control,’ care for mental and behavioral disorders represented 12.8% of all patient consultations in cancer patients evaluated in the telemedicine modality. This means that it is the second cause of consultation in a type of care that is usually accessed by patients who do not present an oncological emergency^5^. The above emphasizes the need to provide comprehensive support to cancer patients, which also involves the psychological and emotional spheres.

On the other hand, Guo *et al*. found that more than a third of women had localized tumors (37%), and only 18.3% were classified as regional. If these data are compared with those reported in Colombia, a slight inversion in the percentages is observed since, in our country, ~50% of women are diagnosed in locally advanced stages and less than a third (27%) in early stages^6^. It is understandable why, in Colombia, ~47% of breast cancer patients had mastectomy^5^. This information makes us wonder if Colombian women with breast cancer have a higher risk of suicide since nearly half had mastectomy as part of their cancer treatment, compared to the general population (Colombian suicide rate in women =2.2 women per 100 000)^2^ and with the rest of the world.

Finally, it is essential to comment that today the oncological outcomes previously considered ‘weak’ are quite relevant. In this sense, research such as that of Guo *et al*. are more than justified since, in light of their findings, it is evident that BCS, whenever possible, should be the preferred surgical option. Unfortunately, in developing countries like Colombia, the diagnosis of breast cancer in locally advanced stages continues to be the norm and BCS is not always feasible.

In conclusion, efforts should be redoubled in order to increase the early diagnosis of breast cancer through mammographic screening, educate health professionals about the importance of evaluating the social determinants related to suicide, treating the medical conditions associated with suicide in a timely manner, and studying other oncological outcomes different than survival, mainly in countries like ours.

## Ethical approval

Not applicable.

## Consent

Not applicable.

## Sources of funding

Not applicable.

## Author contribution

C.B.-M.: conceptualization, investigation, writing, and critical review; X.B.-M.: investigation, writing, and critical review.

## Conflicts of interest disclosure

The authors declare that they have no competing interests.

## Research registration unique identifying number (UIN)

1. Name of the registry: not applicable.
2. Unique identifying number or registration ID: not applicable.
3. Hyperlink to your specific registration (must be publicly accessible and will be checked): not applicable.

## Guarantor

Clara Briceño-Morales and Ximena Briceño-Morales.

## Data availability statement

Not applicable.

## Provenance and peer review

Not applicable.
